# Returning to Town

**Published:** 1859-11

**Authors:** 


### RETURNING TO TOWN.

The weary wanderers after the pleasures and the joys they could not find at home, are now coming back to try it again in the party, the ball, the theatre, the opera, the lecture, the dance and the flirtation. What a hopeless search for happiness! might just as well expect to find a rose growing out of an iceberg.

Some went after the health and vigour and joyousnes, which inexorable "business" had ground out of them, and we hope, and doubt not, that many of our fellow citizens have returned in higher health, and in more buoyant spirit. The object of this article is to suggest how these benefits may be maintained, and how longer enjoyed than they will be if our suggestions are unheeded. The appetite acquires a momentum by change of air and scene, and associations and activities which will carry it on to the first weeks of business, but if that appetite is indulged under circumstances of less pure out-door air, of less exhileration of agreeable and elivening society, with less personal activities, the supply will be greater than the demand, and "fullness" will come, giving head-ache, depression, neuralgia, or "bad-blood," thick, imperfect and impure, will bring nervousness of body, fretfulness of mind, peevishness of disposition, and general irratibility of the whole being, and the sun will cease to shine, clouds will over-cast the clear blue sky of fall, and amid the withering of flowers, hope will die out, and weary, wretched listlessness will, like baleful stars, cast its malign influence over the whole existence.

In truth, it is not an unknown observation, that persons after a joyous and renovating summer, have come home to die, simply because they have eaten with the appetites of an active life, when that activity was no longer observed. Might just as well expect a locomotive not to explode when, although stopped, the fire is still allowed to burn on.

We counsel all then, for the first few weeks after returning to town, to eat one-third less at each meal, until cold weather sets in, when the other third will be required for purposes of fuel, and may be indulged in, not only with impunity, but with pleasure and advantage.

